# Supplementary material for: Comparison of immunogenicity of adjuvanted and high dose influenza vaccination in long-term care facility residents
Source: medRxiv. 2024 Oct 15:2024.10.14.24315459. Preprint. [Version 1] doi: 10.1101/2024.10.14.24315459 (PMC11527040; doi:10.1101/2024.10.14.24315459)

medRxiv preprint doi: https://doi.org/10.1101/2024.10.14.24315459; this version posted October 15, 2024. The copyright holder for this preprint (which was not certified by peer review) is the author/funder, who has granted medRxiv a license to display the preprint in perpetuity. This article is a US Government work. It is not subject to copyright under 17 USC 105 and is also made available for use under a CC0 license.

Supplementary Data

Table S1. Statistical summary of D0 standardized mean difference in outcomes by year and combined. Values below |0.1| were considered well-balanced by randomization, and values exceeding |0.25| were considered strongly unbalanced.

Assay	Strain	2018-2019	2019-2020	Combined
NI	H1N1	-0.19	0.11	-0.02
NI	H3N2	0.01	-0.11	-0.08
HAI	B	-0.13	0.08	-0.01
HAI	H1N1	0.12	-0.08	0.05
HAI	H3N2	0.12	0.03	0.09

medRxiv preprint doi: https://doi.org/10.1101/2024.10.14.24315459; this version posted October 15, 2024. The copyright holder for this preprint (which was not certified by peer review) is the author/funder, who has granted medRxiv a license to display the preprint in perpetuity.
This article is a US Government work. It is not subject to copyright under 17 USC 105 and is also made available for use under a CC0 license.

Table S2. Statistical summary of GMT values for all assays at D0 and D28.

			HD-IIV3				aTIV			
			2018-2019		2019-2020		2018-2019		2019-2020	
**Assay**	**Strain**	**day**	**N**	**GMT (95% CI)**	**N**	**GMT (95% CI)**	**N**	**GMT (95% CI)**	**N**	**GMT (95% CI)**
NI	H1N1	0	83	51.4 (39,67.7)	106	108.1 (77.8,150.2)	89	40.9 (32.2,52)	103	129 (94.3,176.4)
NI	H1N1	28	83	71.8 (53,97.3)	106	297.8 (214.3,413.8)	89	167.7 (128.2,219.3)	103	666.4 (497.7,892.3)
NI	H3N2	0	86	15.3 (13.2,17.8)	106	26.7 (21.9,32.5)	91	15.4 (13.7,17.5)	103	23.8 (19.6,28.9)
NI	H3N2	28	86	24.9 (20.6,30.1)	106	44.7 (35.4,56.4)	91	27.3 (23.3,32.1)	103	46.7 (37.1,58.8)
HAI	B	0	86	56.8 (41.2,78.3)	107	27 (21.2,34.2)	91	47.5 (37.4,60.3)	103	29.8 (23.4,38.1)
HAI	B	28	86	210.4 (152.5,290.3)	107	66.1 (51.1,85.5)	91	113.6 (87.9,146.8)	103	75.5 (58.1,98.1)
HAI	H1N1	0	83	73 (52.7,101)	107	22.4 (18,27.8)	89	87.8 (63.6,121.3)	103	20.5 (16.7,25.2)
HAI	H1N1	28	83	261.9 (192.3,356.6)	107	67.2 (52.3,86.3)	89	213.4 (156.9,290.4)	103	70.5 (53.5,92.9)
HAI	H3N2	0	86	119.7 (89.1,160.8)	107	16.9 (14.2,20)	91	141.6 (103.4,194)	103	17.3 (14.4,20.7)
HAI	H3N2	28	86	517.3 (380.8,702.6)	107	120.4 (87.2,166.2)	91	375.5 (271.3,519.7)	103	140.2 (96.9,202.7)

medRxiv preprint doi: https://doi.org/10.1101/2024.10.14.24315459; this version posted October 15, 2024. The copyright holder for this preprint (which was not certified by peer review) is the author/funder, who has granted medRxiv a license to display the preprint in perpetuity. This article is a US Government work. It is not subject to copyright under 17 USC 105 and is also made available for use under a CC0 license.

Table S3. Statistical summary of HAI and NI seroconversion rates by year.

		HD-IIV3				aTIV			
		**2018-2019**		**2019-2020**		**2018-2019**		**2019-2020**	
**Assay**	**Strain**	**N**	**% Seroconversion (95% CI)**	**N**	**% Seroconversion (95% CI)**	**N**	**% Seroconversion (95% CI)**	**N**	**% Seroconversion (95% CI)**
NI	H1N1	83	9.6 (4.6,18.6)	106	43.4 (33.9,53.4)	89	64 (53.1,73.7)	103	60.2 (50.1,69.6)
NI	H3N2	86	10.5 (5.2,19.4)	106	20.8 (13.7,29.9)	91	20.9 (13.3,30.9)	103	32 (23.4,42.1)
HAI	B	86	47.7 (36.9,58.7)	107	39.3 (30.1,49.2)	91	34.1 (24.7,44.8)	103	36.9 (27.8,47)
HAI	H1N1	83	44.6 (33.8,55.9)	107	43 (33.6,52.9)	89	29.2 (20.3,40)	103	48.5 (38.7,58.5)
HAI	H3N2	86	57 (45.9,67.5)	107	70.1 (60.4,78.4)	91	35.2 (25.6,46)	103	70.9 (61,79.2)

Table S4. Statistical summary of HAI seroprotection rates by year and combined.

		**HD-IIV3**						**aTIV**					
		**2018-2019**		**2019-2020**		**Combined**		**2018-2019**		**2019-2020**		**Combined**	
**day**	**strain**	**Seroprotected/N**	**% (95% CI)**	**Seroprotected/N**	**% (95% CI)**	**Seroprotected/N**	**% (95% CI)**	**Seroprotected/N**	**% (95% CI)**	**Seroprotected/N**	**% (95% CI)**	**Seroprotected/N**	**% (95% CI)**
0	B	54/86	62.8 (51.6,72.8)	42/107	39.3 (30.1,49.2)	96/193	49.7 (42.5,57)	54/91	59.3 (48.5,69.4)	47/103	45.6 (35.9,55.7)	101/194	52.1 (44.8,59.2)
28	B	80/86	93 (84.9,97.1)	77/107	72 (62.3,80)	157/193	81.3 (75,86.4)	79/91	86.8 (77.7,92.7)	80/103	77.7 (68.2,85)	159/194	82 (75.7,87)
0	H1N1	52/83	62.7 (51.3,72.8)	38/107	35.5 (26.7,45.4)	90/190	47.4 (40.1,54.7)	63/89	70.8 (60,79.7)	31/103	30.1 (21.7,40)	94/192	49 (41.7,56.2)
28	H1N1	77/83	92.8 (84.4,97)	76/107	71 (61.3,79.2)	153/190	80.5 (74,85.8)	81/89	91 (82.6,95.8)	75/103	72.8 (63,80.9)	156/192	81.2 (74.9,86.4)
0	H3N2	72/86	83.7 (73.9,90.5)	27/107	25.2 (17.6,34.7)	99/193	51.3 (44,58.5)	77/91	84.6 (75.2,91)	27/103	26.2 (18.3,36)	104/194	53.6 (46.3,60.7)
28	H3N2	83/86	96.5 (89.4,99.1)	85/107	79.4 (70.3,86.4)	168/193	87 (81.3,91.3)	88/91	96.7 (90,99.1)	79/103	76.7 (67.1,84.2)	167/194	86.1 (80.2,90.5)

medRxiv preprint doi: https://doi.org/10.1101/2024.10.14.24315459; this version posted October 15, 2024. The copyright holder for this preprint (which was not certified by peer review) is the author/funder, who has granted medRxiv a license to display the preprint in perpetuity.
This article is a US Government work. It is not subject to copyright under 17 USC 105 and is also made available for use under a CC0 license.

Supplemental Figure 1. HAI and NI GMT titers at D180 for year 1 in figure and table format.

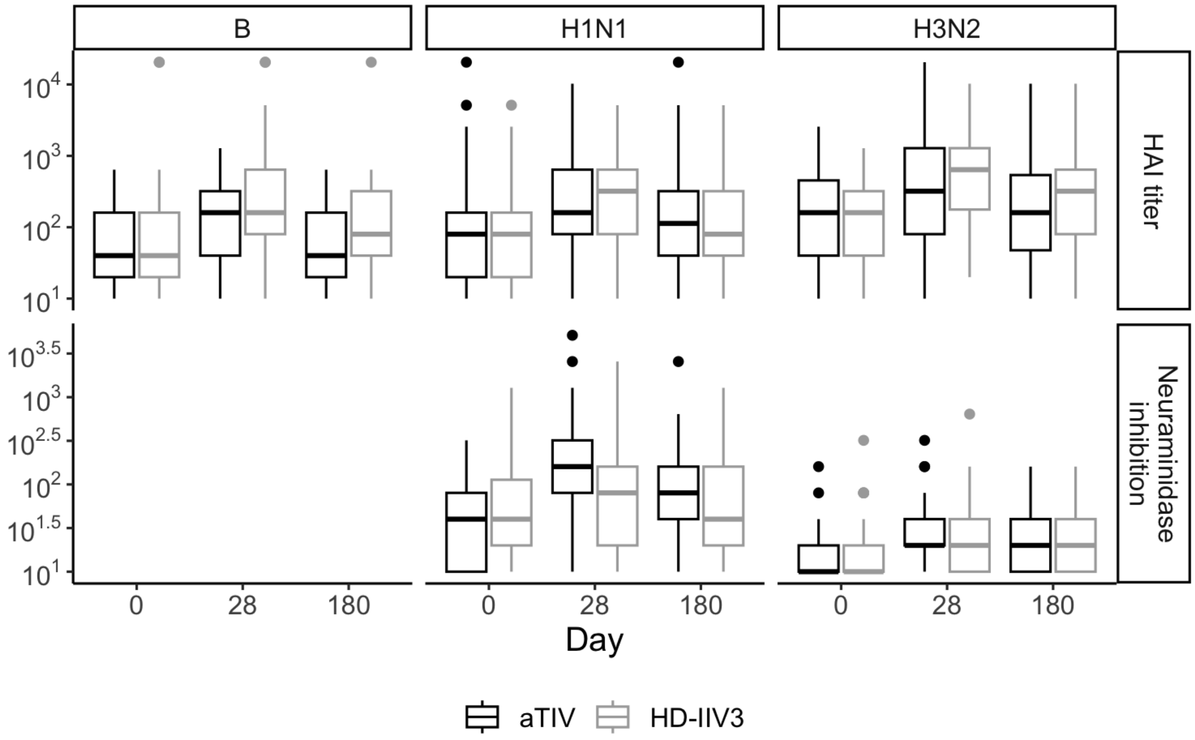

	HD-IIV3		aTIV	
	**N**	**GMT (95% CI)**	**N**	**GMT (95% CI)**
NI, H1N1	73	57.9 (42.5,79)	68	80 (60.1,106.4)
NI, H3N2	72	19.2 (16.2,22.8)	70	20.4 (17.1,24.3)
HAI, B	73	98.6 (72.6,133.8)	70	56 (42.2,74.4)
HAI, H1N1	73	113.2 (80.4,159.4)	68	119.1 (84.2,168.2)
HAI, H3N2	72	228.5 (166.1,314.2)	70	178.4 (123.9,256.8)

medRxiv preprint doi: https://doi.org/10.1101/2024.10.14.24315459; this version posted October 15, 2024. The copyright holder for this preprint (which was not certified by peer review) is the author/funder, who has granted medRxiv a license to display the preprint in perpetuity.
This article is a US Government work. It is not subject to copyright under 17 USC 105 and is also made available for use under a CC0 license.

Supplemental Figure 2. Observed distributions of HAI and NI titers for aTIV, HD-IIV3, and RIV, by day and strain for 2019-2020 season.

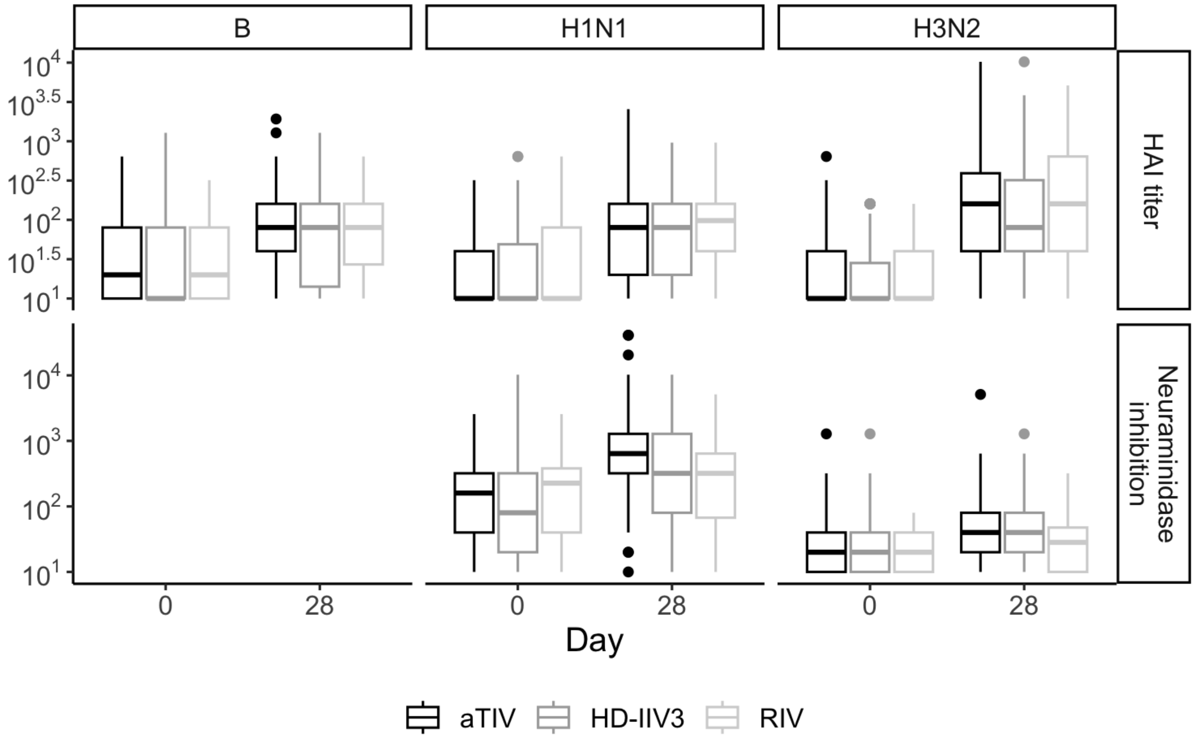

Supplement: Supplement 1 [file NIHPP2024.10.14.24315459v1-supplement-1.pdf]
